# Telepresence Robot Intervention to Reduce Loneliness and Social Isolation in Older Adults Living at Home (Project DOMIROB): Protocol for a Clinical Nonrandomized Study

**DOI:** 10.2196/40528

**Published:** 2022-10-31

**Authors:** Baptiste Isabet, Anne-Sophie Rigaud, Wanji Li, Maribel Pino

**Affiliations:** 1 EA 4468, Faculté de Médecine Université de Paris Paris France; 2 AP-HP, Hôpital Broca Paris France; 3 Institut de Psychologie-Site Boulogne-Université de Paris Boulogne-Billancourt France

**Keywords:** older adults, telepresence robot, feeling of loneliness, social isolation, home, acceptability, usability

## Abstract

**Background:**

There is a growing prevalence of loneliness and social isolation among older adults (OAs). These problems are often associated with depressive states, cognitive decline, sleep disorders, addictions, and increased mortality. To limit loneliness and social isolation in OAs, some authors recommend the use of new communication technologies to maintain a social link with family members as well as with health and social care professionals. Among these communication tools, telepresence robots (TRs) seem to be a promising solution. These robots offer users the possibility of making video calls with their relatives, social workers, and health care professionals, to maintain social contact and access to support services while living at home. Nevertheless, TRs have been relatively unstudied in real-life environments.

**Objective:**

The main objective of this study is to measure the impact of a 12-week intervention using a TR on the feeling of loneliness and on social isolation of OAs living at home. Its secondary objective is to establish recommendations for the implementation of TRs in the studied context.

**Methods:**

A nonrandomized study will be conducted among 60 OAs living at home who will participate in the study for 24 weeks. During this period, they will host a TR for 12 weeks to use it in their home. After the end of the intervention a 12-week follow-up ensues. In total, 4 evaluations will be performed over the entire experimental phase for each participant at weeks 0, 6, 12, and 24. A multidimensional assessment of the impact of the robot will be performed using a multimethod approach including standardized scales and a semistructured interview. This assessment will also help to identify the ergonomic aspects that influence the robot’s usability and acceptability among OAs.

**Results:**

Data collection started in September 2020 and is expected to be completed in early 2023. In August 2022, 56 participants were recruited for the study. Data analysis will take place between August 2022 and is expected to be completed in early 2023.

**Conclusions:**

The DOMIROB study will provide new knowledge on the impact of social TRs in OAs living at home. The results will make it possible to suggest technological, ethical, and organizational recommendations for the use and implementation of TRs for OAs in real-life settings.

**Trial Registration:**

ClinicalTrials.gov NCT04767100; https://clinicaltrials.gov/ct2/show/NCT04767100

**International Registered Report Identifier (IRRID):**

DERR1-10.2196/40528

## Introduction

### The Risk of Loneliness and Social Isolation Among Older Adults

Throughout the world, social isolation and loneliness among older adults (OAs) living at home has reached very high levels. In Europe, in 2018, it was estimated that around 10% of individuals aged over 75 years were socially isolated [1]. In France, in 2017, nearly 300,000 individuals over the age of 60 years experienced social withdrawal [2]. In the United States, one-quarter (25%) of the population over 65 years were socially isolated in 2020 [3]. In Canada, in 2009, 12% of the population over the age of 65 years also encountered this problem [4]. In 2019, it was estimated that about 6 million (7.62%) elderly people lived alone in Japan [5]. However, being socially isolated and experiencing a feeling of loneliness are not the same thing. A person can objectively live in a socially isolated way (ie, having a reduced social network, a lack of social contact or support) and not feel lonely, and conversely, they could not live in a context of social isolation and experience a feeling of loneliness [6]. Loneliness seems to be even more widespread than social isolation among OAs. Several studies estimate that about one-third of OAs experience a feeling of loneliness [7-9], especially after the age of 80 years, with approximately 50% of OAs reporting frequent loneliness [10].

Loneliness and social isolation have particularly harmful consequences for OAs’ psychological and physical health (depression, addictions, cognitive decline, sleep disorders, and excess mortality) [11-15]. Retirement, loss of professional contacts, widowhood, death of relatives and friends, and chronic diseases (which can limit travel) favor withdrawal and social isolation in OAs [16,17].

### New Technologies: A Socialization Solution for OAs?

Several authors have advocated interventions using technologies, such as computers, tablets, the internet, SMS text messaging and social media apps, videoconferencing, or robots, to alleviate loneliness and improve quality of life in OAs [18,19]. Digital technologies (eg, tablets, social networks, video games, and robots) can be useful to mitigate the consequences of social isolation and loneliness in OAs [19-23]. Digital tools allow users to get information through the internet, to maintain social contact with their relatives, and to improve social participation via different communication tools (eg, instant messaging and social networks). Nevertheless, the digital divide persists, notably because these technologies are not tailored to OAs’ needs and preferences. It is important that the technology is ergonomic, easy to use, and useful for this population. For example, it is recommended to use simplified interfaces and contents that meet OAs’ capacities and preferences in addition to introducing tools (eg, stands, tablet holders, stylus) that can increase the comfort and ease of use of the equipment for the individual [24]. Today, different actors from the field of innovation and digital technologies work on the development of adapted solutions to meet OAs’ needs that could minimize the digital divide. Telepresence robots (TRs) are an example. TRs are tools that help create a connection between 2 distinct environments through cameras, microphones, speakers, and a screen. TRs offer a videoconferencing functionality integrated on a navigation base with wheels that allow the robot to move around in an environment. TRs can be used to promote social contact with relatives and friends and access to a wide range of distant assistance services. Although TRs seem to have promising functionalities for the management of isolation in OAs, they remain poorly studied and deployed in home environments.

### TRs: A Solution That Has Not Been Thoroughly Studied for Seniors Living at Home

Since the beginning of the COVID-19 health crisis, different stakeholders have shown an increasing interest in innovative remote communication solutions and TRs [25-38]. However, thus far, there is limited available evidence concerning the implementation and usefulness of TRs in a home-dwelling OA population. TRs have so far been studied mainly in controlled settings [39-49], although some studies involving OAs have been conducted in institutional settings (hospital services or senior residences) [50-58] and a few of them at home [59-63]. In particular, studies conducted with OAs at home have highlighted the benefits and issues associated with the use of TRs.

With regard to the benefits of using TRs at home, cognitively healthy users have had a positive experience [60-63]. Participants in the experiments reported good acceptance, usability, perceived usefulness [59], and reliability of the TRs [63]. In the study by Cesta et al [43] the users mentioned good social and functional acceptability of the robot without any loss of interest in its use over time. They considered the mobility, entertainment, and obstacle detection features of the robot to be satisfactory [61]. They mentioned that TRs could be beneficial for physical health, psychological well-being, social contact, and independent living [60]. In the trial carried out by Bakas et al [62], OAs showed an improvement in quality of life and sleep, as well as a decrease in depression following a TR intervention.

Elderly participants of TR studies have also mentioned some issues when experimenting with these tools at home. In the study by Gonzalez-Jimenez et al [59], some participants feared that video calls with TRs might replace real human contact with relatives and friends. They also noted that the robot was too big and too noisy and that its battery required too much energy. In another study [60], the obstacles identified to the implementation of TRs were the unsuitability of the robot’s wheels for different types of floors and a slight confusion for some users when using the handheld remote control. Concerns about operating the robot from a distance were also reported by some secondary users (eg, family members or friends). One participant with mild cognitive impairment requested the withdrawal of the robot due to the significant difficulties encountered while using it. Therefore, the authors concluded that TRs were not suitable for OAs with mild cognitive impairment. Participants also emphasized the need for good speech recognition, navigation, and self-location of TRs [61] in addition to a good internet connection to optimize their use [59,63].

Although these studies reported interesting results regarding the implementation and acceptability of TRs in OAs homes, some methodological limitations were identified, such as small sample sizes included in the studies (between 2 and 20 participants), and the fact that in some protocols the TR was tested using different implementation periods for different users within the same sample, which does not allow to have the same frequency of use of the TR and may affect its impact. Besides, the psychosocial effects of the TR intervention (eg, including outcomes such as depressive symptoms or quality of life) was measured in only 1 study [61].

To overcome the aforesaid limitations identified in the literature, the DOMIROB project aims to implement a TR and assess its impact in 60 OAs living at home for a period of 12 weeks, with the same length of implementation for all participants. The main objective of this study is to measure the impact of a TR intervention on the feeling of loneliness and social isolation of OAs living at home who may benefit from the robot for a 12-week period. We hypothesize that the use of a TR at home would reduce the feeling of loneliness and social isolation in OAs.

The experimental protocol of the DOMIROB project, inspired by the MARTA (Multidimensional Assessment of Telepresence Robot for Older Adults) model [59], allows the psychosocial and ergonomic dimensions of TRs to be examined using a multimethod longitudinal design. To avoid the risk of complex and cumbersome evaluation for the users, we have reduced the number of evaluation scales in the MARTA model and have only kept the measures for loneliness, perceived social support, depression, acceptability, usability, self-perceived health, and the effect of a device on independent living. Concerning the qualitative evaluation of the experimentation, we propose a complementary and original assessment performed using a semidirective interview inspired by the Core Model of the European Network for Health Technology Assessment (EUnetHTA) [64], which is described later.

## Methods

### Study Design

The DOMIROB protocol is a nonrandomized, quasi-experimental field trial using a multimethod and multidimensional assessment. The experimental phase of the study took place between September 2020 and February 2023. Volunteer OAs were recruited between September 2020 and August 2022 in Paris, France. This study included 60 OAs who agreed to host and use a TR in their home for a period of 12 weeks.

### Determination of Sample Size

The sample size and power calculation for this study were performed based on 1 of the main outcome measures for this study: the feeling of loneliness, as assessed with the Perceived Loneliness Scale (UCLA; version 3) [65]. From a statistical point of view, we consider that the average score for the UCLA scale, observed for people aged 65 years and over, is 31.51/80 [65]. The higher the score, the greater the perceived loneliness is evaluated. Therefore, our hypothesis aims at a decrease in the average score in the UCLA scale of 3-5 points. If we want to show a decrease of 15% in the average score on the UCLA, between the assessment done at week 0 and week 24 with a power of 0.8 and a risk α of .05, then 60 individuals should be included. A decrease in the UCLA loneliness score of 15% after a 12-week implementation of the TR is clinically relevant.

### Objectives

#### Main Objective and Primary Outcome Measures

The main objective of this study is to measure the impact of a 12-week intervention using a TR in the home of OAs on their feeling of loneliness and social isolation. To measure the impact of TRs on the feeling of loneliness, we will use the UCLA (version 3) [65]. We will assess social isolation using the Multidimensional Scale of Perceived Social Support (MSPSS), which is a tool for measuring the perception of social support that a person has [66]. We will compare the results obtained in the assessments performed at weeks 0, 6, 12, and 24 to identify the possible decreases in the scores of the different scales at different times of the implementation of the TR at home.

#### Secondary Objectives and Secondary Outcome Measures

The secondary objective of this study is to establish recommendations for the implementation of TRs in the homes of the OAs. The goal is therefore to study the use of TRs over 12 weeks using ergonomic and health-related and psychosocial criteria. The ergonomic parameters include an evaluation of the perceived usability of the robot software (System Usability Scale [SUS]) [67] and the acceptance of the robot (ALMERE model) [68]. The psychosocial parameters include an evaluation of depressive states (Geriatric Depression Scale [GDS]) [69] and the psychosocial impact of the robot (Psychosocial Impact of Assistive Devices Scale [PIADS]) [70]. These secondary outcome measures are assessed at weeks 6 and 12 (Table 3).

These assessments will help us determine whether TRs are useful tools to reinforce social contact for OAs living at home. We will identify the most suitable target population (eg, autonomous OAs, dependent OAs) as well as the most suitable framework and environment of use (eg, home, senior residence) for the deployment of TRs. We will also suggest organizational, ethical, and practical recommendations for the implementation and use of TRs by community-dwelling OAs.

### Participants

The recruitment of volunteer participants is carried out through the outpatient clinic of the Parisian geriatric hospital (Broca Hospital, Assistance Publique–Hôpitaux de Paris) and its network of professionals, “seniors” associations, and town halls in the Paris region. The inclusion and exclusion criteria for participation in the study are described in Textbox 1.

**Figure 1:**
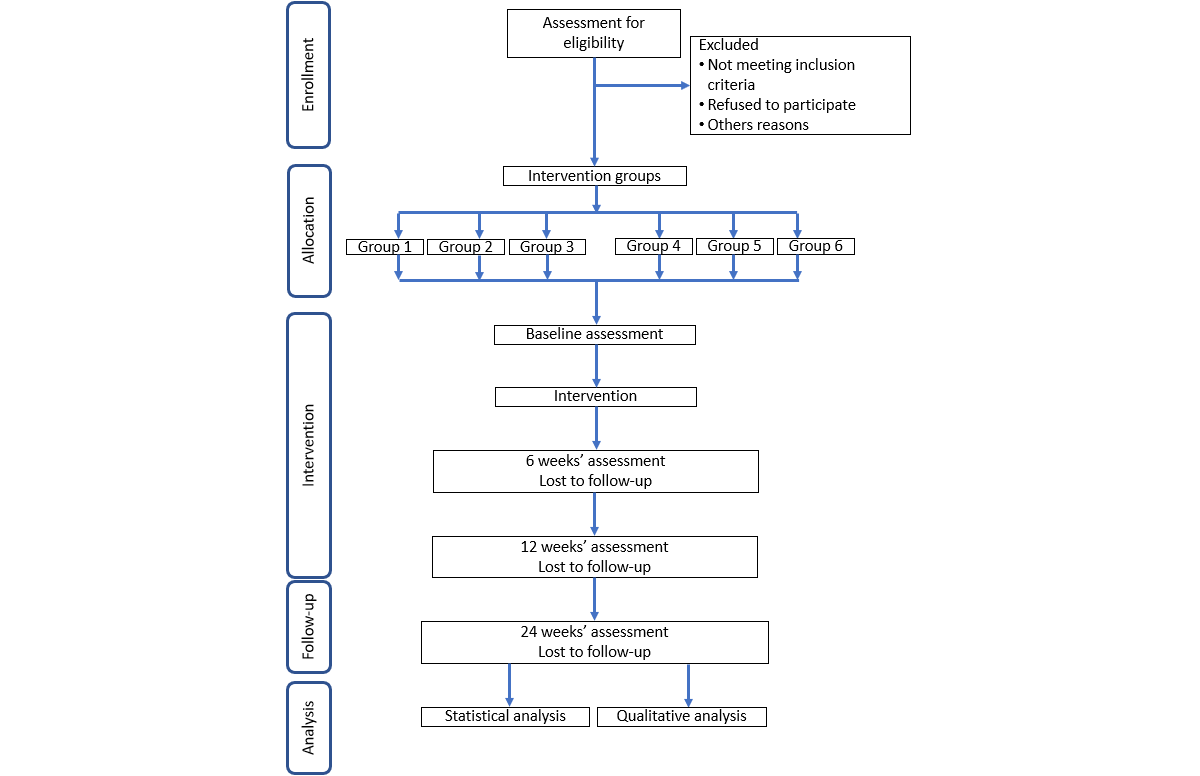
Flow chart of the recruitment process.

Information leaflets are given to professionals working in the outpatient hospital of Broca Hospital to distribute them to potential volunteers during consultations. Senior citizens’ associations also distribute the information leaflet to their members and the local city halls disseminate a communication about the study to their users. When OAs wish to volunteer to take part in the trial, they contact the researcher in charge of the study to discuss the modalities in more detail, either by email or by telephone, using the information given on the information leaflet. During this first contact, a detailed explanation of the study is given. After having obtained answers to all their questions, volunteers receive an email with an information note describing the whole study at least 24 hours before inclusion, to give them time to withdraw from participation if wished. Participants are divided into 6 groups of 10 people; 10 robots were available simultaneously. A group of 10 participants was formed every 12 weeks. Each participant who agrees to participate is invited to sign the study’s no-opposition form. The recruitment procedure is described in Figure 1.

**Inclusion and exclusion criteria for the DOMIROB study.**

**Inclusion criteria**

- Being 65 years of age and over;
- living in the Paris region (Île-de-France);
- express nonopposition to participating in the study;
- agree to host a telepresence robot in the home for 12 weeks; and
- having an internet connection at home.

**Exclusion criteria**

- Being under 65 years of age;
- expressed opposition to participating in the study;
- having moderate or major neurocognitive disorders (Mini-Mental State Examination score <20; [71]);
- being under guardianship or curatorship; and
- living in housing unsuitable for the telepresence robot (surface area and configuration of the home not suitable for robot circulation).

### Ethics Approval and Consent

The research protocol was submitted to, and approved by, a French national institutional review board for research involving human participants (Comité de Protection des Personnes, France) on April 27, 2020 (national number: 2020-A00381-38. Trial registration: ClinicalTrials.gov, trial registration number NCT04767100).

### Materials

**Figure 2:**
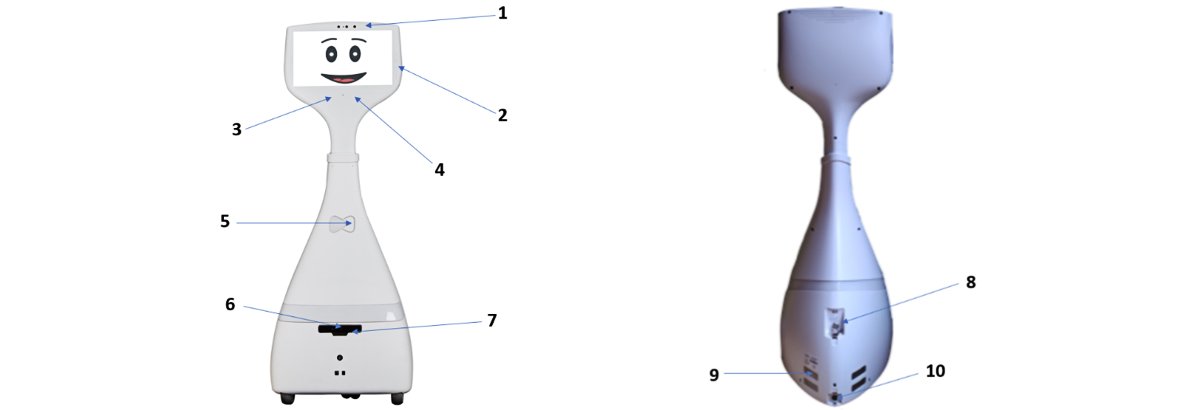
Front and back view of the telepresence robot Cutii. Note: 1: video call camera; 2: touch screen; 3: speaker; 4: microphone; 5: battery indicator light; 6: navigation camera; 7: obstacle detector; 8: power switch; 9: port for dock charging; 10: port for charging by charger.

The TR used in this study is the Cutii, a robotic platform for remote communication. This TR is equipped with a touch screen, microphones, loudspeakers, cameras, an obstacle detector, and a mobile system allowing navigation in the environment (Figure 2). Cutii allows users to maintain a connection with their environment. Its interface has different functionalities allowing, for example, to make video calls and to participate in intellectual (eg, virtual museum tour) and physical (eg, yoga) stimulation activities. Cutii’s services include the following:

- An agenda with a calendar to schedule the activities.
- A contact directory for video calls.
- An “activities” tab allowing users to participate in collective live sessions of intellectual and physical activities/workshops (eg, gymnastics, yoga, art therapy, virtual museum tours) led by professionals through the video call feature.
- A “leisure” tab allowing users to play digital games (eg, memory games, sudoku, quiz).
- A “video” tab to watch documentaries (eg, cooking, traveling, animals’ life).
- A “teleconsultation” tab that allows users to consult a health professional remotely through the video calling system.

### Measures

We will use a multimethod approach assessment protocol partly inspired by the MARTA model [43], which has been developed to assess TR interventions involving OAs and complemented by the EUnetHTA model. The assessment uses standardized scales and a semistructured interview format (Multimedia Appendix 1). Sociodemographic data of the participants are also collected (age, sociocultural level, lives alone or accompanied, has children/grandchildren or not) as well as their Mini-Mental State Examination (MMSE) [72] score, which measures the global cognitive functioning, and the 12-Item Short-Form Health Survey (SF-12) [73] score, which measures the perception of one’s health status.

### Standardized Scales

The MMSE [72] measures the global cognitive functioning of OAs. MMSE scores range from 0 to 30, with scores of 26 or higher being considered normal (ie, absence of cognitive impairment). Moderate or major neurocognitive disorders are considered to be present when the score is below 20 [66].

The UCLA (version 3) [65] measures the feeling of loneliness in participants with 20 questions. Participants answer the questions using a Likert scale consisting of 4 response choices: “1=never,” “2=rarely,”, “3=sometimes,” and “4=always.” The scoring of this scale is based on the sum of the scores obtained for each item. The score correlates directly with loneliness, that is, the higher the score, the more lonely the participant feels.

The MSPSS [66] measures the social support perceived by a person. This 12-item scale is divided into 3 subscales: “family,” “friends,” and “others.” Participants respond to each statement using a Likert scale ranging from “1=totally disagree” to “7=totally agree.” The global score allows the researcher to identify 3 levels of perceived social support: high (61-84), moderate (36-60), and low (0-35).

The 30-item GDS [69] measures depressive states in a geriatric population. It is composed of 30 items to which participants answer “yes” or “no.” The total score is calculated by assigning a “0” or a “1” to each item according to the participant’s response. The highest score is therefore 30. A score ranging from 0 to 9 represents a normal state. A score from 10 to 19 or from 20 to 30 represents, respectively, a moderate or severe depression.

The SF*-*12 [73] is a questionnaire developed from the SF-36 questionnaire. The SF-12 provides a self-reported measure of the impact of physical and mental health in the everyday life of individuals. The average score is 50 and all scores above this threshold are considered normal.

The SUS [67] defines the level of usability of a system, tool, software, or digital technology with regard to its effectiveness, efficiency, and overall ease of use. After having experienced the use of the device, the person responds to 10 items using a Likert scale consisting of 5 responses ranging from “1=strongly disagree” to “5=strongly agree.” The total score ranging from 0 to 100 represents a gradually increasing usability. A product is considered as having a good usability if the score is above 70.

The ALMERE model [68] measures OAs’ acceptance toward socially assistive robots. It can be used to predict and understand the use of a system by observing the influences on the intention to use it. The ALMERE model questionnaire is composed of 39 items divided into 13 dimensions: “Anxiety,” “Attitude Towards Technology,” “Facilitating Conditions,” “Intention to Use,” “Perceived Adaptiveness,” “Perceived Enjoyment,” “Perceived Ease of Use,” “Perceived Sociability,” “Perceived Usefulness,” “Social Influence,” “Social Presence,” “Trust,” and “Use.” These dimensions predict the actual intention to use the system. To answer the items, the participants respond using a 5-point Likert scale ranging from “1=strongly disagree” to “5=strongly agree.” Scores of 1 and 2 represent poor satisfaction, 3 denotes fair satisfaction, 4 means good satisfaction, and 5 promotes excellent satisfaction.

The PIADS [70] is a self-report questionnaire designed to assess the effects of an assistive device on functional independence, well-being, and quality of life. Participants respond to 26 items on a 7-point Likert scale ranging from “–3” to “+3.” The final score of –3 represents the strongest negative impact, 0 indicates no perceived impact, and 3 denotes the strongest positive impact.

Measurements performed during the experimental phase of the DOMIROB study are listed in Table 1. The calendar of the assessments is specified in Table 2.

**Table 1:** DOMIROB project protocol measures.

Measurement tools	Dimensions assessed
Mini-Mental State Examination (MMSE) [72]	Global cognitive efficiency
Perceived Loneliness Scale (UCLA) [65]	Subjective feelings of loneliness
Multidimensional Scale of Perceived Social Support (MSPSS) [66]	Perceived social support (social isolation)
Depression: Geriatric Depression Scale (GDS) [69]	Depressive states
Perceived health status: 12-item Short Form Health Survey (SF-12) [73]	Self-assessment of health
System Usability Scale (SUS) [67]	Telepresence robot usability
Acceptance model (ALMERE) [68]	Telepresence robot acceptance
Psychological Impact of the Assisted Device (PIADS) [70]	Psychosocial impact of a device
Sociodemographic data	Age, socioeducational level, and family status
Semistructured interview	Eight dimensions of the European Network for Health Technology Assessment Core Model: “Health Problem and Current Use of the Technology,” “Description and Technical Characteristics of the Technology,” “Safety,” “Clinical Effectiveness,” “Costs and Economic Evaluation,” “Ethical Analysis,” “Organizational Aspects,” “Patients and Social Aspects” [65]

**Table 2:** Semistructured interview based on the European Network for Health Technology Assessment Model.

Item	Health Technology Assessment dimension
What impact did the robot have on your health/well-being?	Current Use of the Technology (CUR)
How did you find the robot’s features and services? Did you find them useful? Why do you think so?	Description and Technical Characteristics of the Technology (TEC)
What do you think are the potential risks and side effects caused by the use of the robot? What can be done to prevent them?	Safety (SAF)
Do you think this robot can have an impact on the loneliness/isolation of the users? Why?	Clinical Effectiveness (EFF)
How much would you be willing to invest to benefit from the robot in your home? Would you prefer to purchase or to rent the robot? In the case of a rent, would it be for a short or long term?	Costs and Economic Evaluation (ECO)
In your opinion, what are the ethical issues to be identified and defined before the deployment of these robots in the homes of future users?	Ethical Analysis (ETH)
In your opinion, what skills and knowledge are necessary for a good deployment of these robots in users’ homes?	Organizational Aspects (ORG)
What factors would restrain you from using this type of robot?	Patients and Social Aspects (SOC)
Nonapplicable	Legal Aspects (LEG)

### Semistructured Interview

We designed a semistructured interview based on the EUnetHTA Core Model version 3.0 [65]. This model allows a systematic assessment of the characteristics, effects, or impacts of health care technologies. The main objective of the HTA model is to facilitate decision making in the field of health care to improve the uptake of new health technologies.

The 9 dimensions of the EUnetHTA Core Model allow the identification of issues that are necessary for the deployment and use of new technological tools in the field of health. These dimensions include “Health and Current Use of the Technology,” “Description and Technical Characteristics of the Technology,” “Safety,” “Clinical Effectiveness,” “Costs and Economic Evaluation,” “Ethical Analysis,” “Organizational Aspects,” “Patients and Social Aspects,” and “Legal Aspects.” The interview guide used in the DOMIROB protocol was designed using the first 8 dimensions of the EUnetHTA Core Model (Table 2). The “Legal” dimension was excluded because its assessment did not directly concern the TR end users and was therefore entrusted to a specialized consulting firm. The semistructured interviews were integrated to our protocol with the aim of examining different dimensions that may inform the choice of TRs to provide social and care services to OAs and to establish recommendations for the use of TRs for future users.

### Procedure and Time Schedule

**Figure 3:**
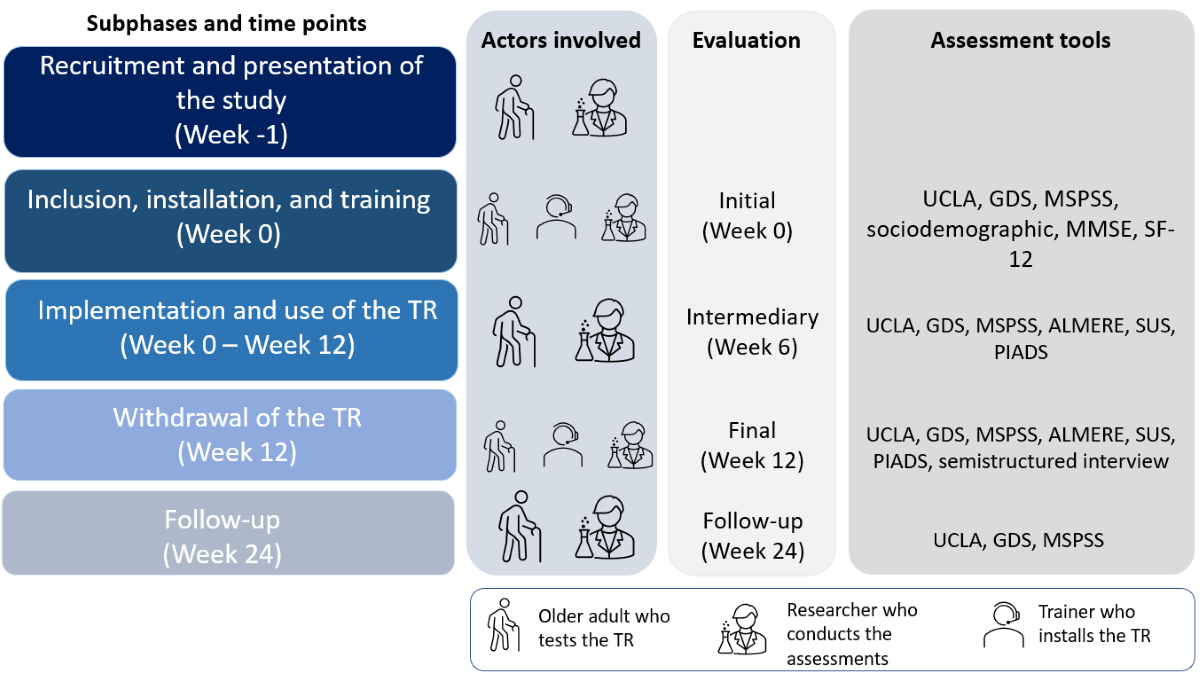
Steps of the experimental phase. GDS: Geriatric Depression Scale; MMSE: Mini-Mental State Examination; MSPSS: Multidimensional Scale of Perceived Social Support; PIADS: Psychosocial Impact of Assistive Devices Scale; SF-12: 12-Item Short-Form Health Survey; SUS: System Usability Scale; UCLA: Perceived Loneliness Scale.

All stages of the experimental phase are illustrated in Figure 3, which describes the different actors involved in each step of the experimental phase, its length, the eventual assessment carried out at that moment, and the respective assessment tools.

During their participation in the protocol, the participants complete 4 assessments (including scales and interviews) as shown in Table 3.

**Figure 4:**
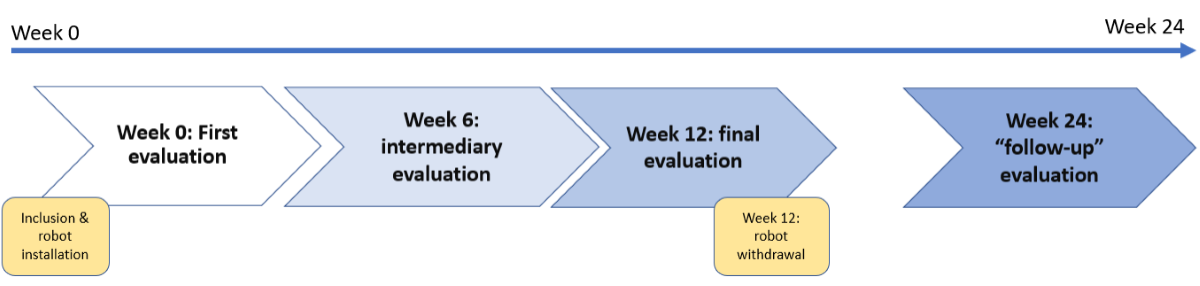
Experimental and evaluation phases in the DOMIROB protocol.

The first evaluation is performed at the time of inclusion at week 0 (ie, approximately 1 week before the implementation of the TR in the volunteers’ homes; Figure 4). A second evaluation (intermediate evaluation) is conducted 6 weeks after the beginning of the experiment at home. A third evaluation (final evaluation) is carried out at the end of the implementation phase (week 12). To conclude the participation in the protocol, the volunteers are invited to take part in a final assessment (follow-up evaluation) at week 24, approximately 12 weeks after the robot has been removed from the participants’ homes. Assessments of participants are conducted face-to-face for the inclusion, and then via the video call functionality of the Cutii robot or by telephone at weeks 6, 12, and 24. Semistructured interviews at week 12 are conducted by a psychologist and recorded using a voice recorder to be transcribed and analyzed.

**Table 3:** Calendar of the assessment carried out in the DOMIROB protocol.

Measures	Evaluations			
	Inclusion (week 0)	Week 6	Week 12	Week 24
Mini-Mental State Examination (MMSE)	✓			
Perceived Loneliness Scale (UCLA)	✓	✓	✓	✓
Multidimensional Scale of Perceived Social Support (MSPSS)	✓	✓	✓	✓
Geriatric Depression Scale (GDS)	✓	✓	✓	✓
12-Item Short-Form Health Survey (SF-12)	✓			
System Usability Scale (SUS)		✓	✓	
The ALMERE model		✓	✓	
Psychosocial Impact of Assistive Devices Scale (PIADS)		✓	✓	
Sociodemographic data	✓			
Semistructured interview (Health Technology Assessment model)			✓	

### Installation and Training

For the implementation phase of the robot, a team of 2 professionals, experts of the Cutii robot, install the robot in the participant’s home. The team and the participant choose the location of the dock of the robot, which is close to an outlet to facilitate its recharging. During the intervention, the team connects the robot to the participant’s Wi-Fi network and ensures it works properly. After the installation, the team provides the participants with a 60-minute training session to familiarize them with its functionalities and operation. The training session includes information about how to use the robot, its maintenance, and the procedure to follow in case of malfunction. In case of malfunction, participants can contact the project support team at any time during the study. Participants are informed that their environment can be visualized during the video call service and the group workshops so that they can make the arrangements when necessary to preserve their privacy.

### Period of Use of the Robot

During the experimental phase, participants are invited to discover the different functionalities of the robot. Participants are free to use the TR as needed and wished. Volunteers can discover the “video call” functionality by making videoconferences with their relatives or taking part in the live sessions (workshops, activities). Every week the workshop facilitators offer live cultural and physical workshops (eg, yoga, soft gym, guided tours of museums) through the robot’s “video call” feature. The robot also offers users games, reading, and music applications.

### Data Analysis

#### Quantitative Analysis (Standardized Scales)

The descriptive data for all the questionnaires will be presented in the form of both average scores and SDs. Inferential statistical analyses of the results obtained during the different evaluation weeks will then be performed. All collected data will be analyzed using the open-source statistics program Jeffreys’s Amazing Statistics Program (version 0.14.1; JASP Team). For the scores of questionnaires on social isolation (GDS, MSPSS, and UCLA), a 1-way ANOVA or its nonparametric alternative (the Friedman test) will be applied according to the result of Shapiro-Wilk normality test. Then the Mauchly test of sphericity will be performed to see if any postcorrection of the degrees of freedom has to be made so that the valid *F*-ratio can be obtained. For the questionnaires concerning the use of TRs (SUS, PIADS, and ALMERE), the Shapiro-Wilk test will be used to verify the assumption of normal distribution of data to determine whether the paired (2-tailed) sample *t* test or Wilcoxon signed-rank test will be used. It should be noted that for a better interpretation of the results of the ALMERE, the Cronbach α index will be used to ensure a proper internal consistency of the questionnaire. Similarly, the multiple linear regression will be used to determine the predictive links between different aspects presented in the ALMERE model.

#### Qualitative Analysis (Semistructured Interviews)

For the analysis of semistructured interviews, we used a framework analysis approach [74], a thematic analysis that allows one to identify a qualitative data set of deductively derived themes (based on the dimensions directly addressed in the interview guide, in our case the EUnetHTA Core Model dimensions) and inductively derived themes (newly emergent data). The combination of deductively and inductively derived themes constitutes the framework. This approach has the advantage of allowing the identification, description, and analysis of qualitative data in an efficient way with certain flexibility. To conduct this framework analysis, the interview recordings are listened to and transcribed to have a comprehensive and global understanding of the participants’ answers. We then identify the themes related to the dimensions of the EUnetHTA Core Model [64] (eg, “Health Problem and Current Use of Technology,” “Description of the Technology and Technological Characteristics,” “Safety”), which are linked to the questions administered to participants during the interview. The identification of more abstract concepts follows, with the aim of creating the framework for the analysis. In this study, this refers to the regrouping or ranking of the aspects or arguments that can facilitate or hinder the implementation or the adoption of an intervention with TRs in the OAs’ home. Then, in the indexing stage, where the transcripts are classified according to the framework, verbatims are labeled with codes and grouped under the corresponding categories of the framework. Finally, all the themes are listed in a Microsoft Excel table with different subcategories as well as their corresponding verbatim and labels. Finally, by discovering the patterns in the data and identifying the similarities, the results are interpreted.

## Results

Recruitment will end in August 2022. A total of 56 participants have been recruited into the study. Analysis of the results started in August 2022 and its completion is expected at the beginning of 2023.

## Discussion

### Expected Findings

The main objective of the DOMIROB project is to measure the impact of TRs on social isolation and loneliness in OAs living at home. As far as the benefits of the trial are concerned, we expect that the TR intervention improves the feeling of loneliness and social isolation of OAs as suggested by Troen [75]. Cesta et al [43] showed that TRs can bring a better sense of well-being with a boost in self-esteem and a decrease in social isolation in an aging population. For our study, we begin with the premise that the implementation of a TR could provide participants with new social contacts, a feeling of social belonging, and a strengthening of already existing social ties. These actions should translate into a decrease in the feeling of loneliness and social isolation as well as anxiety-depressive states measured by standardized scales.

So far, there are little data on the impact of TR implementation in community-dwelling persons. Available results in the literature mainly concern exploratory studies that included a limited number of participants, used short intervention periods, and experimental protocols that evaluated only 1 or 2 impact dimensions. The DOMIROB protocol, designed and adapted from the MARTA model [43], proposes a multidimensional assessment to study the psychosocial and ergonomic outcomes of the TR intervention using a multimethod approach. This study will evaluate the impact of TRs in a sample of OAs in an ecological situation over a similar length of use of the TR for all participants.

The use of the MARTA model was chosen because the study conducted by Cesta et al [43] has many similarities with the DOMIROB project. Both studies aimed to evaluate the usability and acceptability of TRs in an ecological setting with an elderly population. The adaptability of the MARTA model allows us to define a protocol that meets our objectives. As our main aim is to measure the impact of TRs on social isolation in OAs living at home, we chose to keep the psychosocial scales of the MARTA model, although not all of them were used in the study by Cesta et al [43]. Further, 2 participants included in this study lived together. Two scales, namely, the Temple Presence Inventory (TPI) [76] and the Positive and Negative Affect Schedule (PANAS) [77], were excluded from the DOMIROB project to reduce the number of assessment tools and to limit fatigue and cognitive overload in OAs during the evaluations. Indeed, the Cesta et al [43] study was carried out over a period of 12 months with only a couple of OAs, whereas the DOMIROB project aims to recruit 60 participants, with each volunteer participating in the study for 6 months during a total experimental period of 18 months.

The DOMIROB project is a truly comprehensive study on the implementation of TRs for OAs living in home, particularly regarding the psychosocial impact of TRs on OAs in home. It is also the first study to include a multidimensional assessment of both psychosocial and ergonomic aspects. This trial will allow the identification of OAs’ profiles for whom the implementation of TRs seems the most relevant. We expect that TR implementation for OAs at home could contribute to limit their social withdrawal. However, it is also conceivable that TRs fail to meet the main aim of the intervention, that they are unsuitable for the users’ homes, or that their use and maintenance require significant help, thus limiting their interest for OAs who cannot regularly benefit from the assistance of a third person.

The results of this study will contribute to not only the development of recommendations for the use and development of educational tools, but also for health professionals to integrate these tools into their practice, to identify facilitating factors and the organizational and ethical constraints related to the implementation of TRs in OAs’ homes. Results from the qualitative analysis (semistructured interview), based on the EUnetHTA Core Model, will help to identify the different impacts of TRs on the health and daily life of users and to identify some socioeconomic issues related to the implementation of these new tools. At the end of the experimental phase, usage, ethical, and organizational recommendations will be established to design a users’ guide.

### Study Limitations

This study has several limitations. First, we did not consider having a confirmed feeling of loneliness or being socially isolated an inclusion criterion for the recruitment of participants, which could limit the impact of the intervention. However, we would like to emphasize that as shown in the literature [7-10], loneliness and social isolation are frequently observed in this age group. Second, the persons recruited for this study were required to have an internet connection at home. This inclusion criteria may induce a bias because OAs who are already internet users may have a more positive view of digital technologies, such as TRs, and be more familiar with them, than people who do not have internet at home. Third, all the volunteers are recruited in Ile-de-France (ie, Paris and its suburbs). Thus, one cannot exclude the possibility that OAs living in other environments (eg, in rural areas) might show an acceptance of robots different from that of OAs living in more urban areas. Fourth, the experimental group was not compared with a control group because of logistical constraints. Fifth, participants in this protocol tested the robot at home in different contexts and periods of the year (eg, lockdown period linked to COVID-19, summer or winter periods, holiday or working periods). Therefore, one cannot exclude the fact that the use of TRs might be different according to the context when it is tested by OAs. Finally, one could discuss the test-retest reliability of the scales (eg, UCLA, MSPSS, and GDS), considering that the authors administer them once every 6 weeks. For example, the UCLA has a test-retest reliability of 1 year. Therefore, using this tool once every 6 weeks could be a potential limitation of this study.

### Conclusions

The DOMIROB project aims to measure the impact of TRs on the feeling of loneliness and social isolation in OAs living at home and to establish practical, ethical, and organizational recommendations for the use of these new tools.

## Appendix

Multimedia Appendix 1 Assessment and interview tools used during the experimental phase.

## Abbreviations

EUnetHTA: European Network for Health Technology Assessment
GDS: Geriatric Depression Scale
MARTA: Multidimensional Assessment of Telepresence Robot for Older Adults
MMSE: Mini-Mental State Examination
MSPSS: Multidimensional Scale of Perceived Social Support
OA: older adult
PANAS: Positive and Negative Affect Schedule
PIADS: Psychosocial Impact of Assistive Devices Scale
SF-12: 12-Item Short-Form Health Survey
SUS: System Usability Scale
TPI: Temple Presence Inventory
TR: telepresence robot
UCLA: Perceived Loneliness Scale

## Data Availability

The data sets generated during or analyzed during the current study are available from the corresponding author on reasonable request.
