# Supplementary material for: Telepresence Robot Intervention to Reduce Loneliness and Social Isolation in Older Adults Living at Home (Project DOMIROB): Protocol for a Clinical Nonrandomized Study
Source: JMIR Res Protoc. 2022 Oct 31;11(10):e40528. doi: 10.2196/40528 (PMC9664327; doi:10.2196/40528)
Supplement: Multimedia Appendix 1 [file resprot_v11i10e40528_app1.docx]

**Appendix 1:** Assessment and interview tools used during the experimental phase

Socio-demographic data:

Date of birth:

NSC:

Child(ren)/grandchild(ren):

Lives (alone/accompanied):

Quantitative evaluation:

The scales used in this protocol are subject to copyright. Here are the references where they can be found.

*The Mini Mental State Examination* (MMSE)

Reference: Kalafat M, Hugonot-Diener L, Poitrenaud J. Standardisation et étalonnage français du “Mini Mental State” (MMS) version GRECO. Revue de neuropsychologie. 2003;13(2):209-236.Ware JE, Kosinski M, Keller SD. A 12-Item Short-Form Health Survey: Construction of Scales and Preliminary Tests of Reliability and Validity. Medical Care. 1996;34(3):220-233.

*The UCLA Loneliness Scale* (*UCLA*, version 3)

Reference: Russell DW. UCLA Loneliness Scale (Version 3): Reliability, Validity, and Factor Structure. Journal of Personality Assessment. 1996;66(1):20-40. [doi:10.1207/s15327752jpa6601_2]

The *Multidimensional Scale of Perceived Social Support (MSPSS*)

Reference: Zimet GD, Dahlem NW, Zimet SG, Farley GK. The Multidimensional Scale of Perceived Social Support. null. 1988;52(1):30-41. [doi:10.1207/s15327752jpa5201_2]

*The Geriatric Depression Scale 30 item* (*GDS-30*)

Reference: Yesavage JA, Brink TL, Rose TL, et al. Development and validation of a geriatric depression screening scale: A preliminary report. Journal of Psychiatric Research. 1982;17(1):37-49. [doi:10.1016/0022-3956(82)90033-4.]

The *12-Item Short Health Survey (SF-12*)

Reference: *Ware JE, Kosinski M, Keller SD. A 12-Item Short-Form Health Survey: Construction of Scales and Preliminary Tests of Reliability and Validity. Medical Care. 1996;34(3):220-233.*

*The System Usability Scale (SUS*)

Reference: Brooke J. SUS - A quick and dirty usability scale. :8. [ISBN: 9780429157011]

*The ALMERE model*

Reference: Heerink M, Kröse B, Evers V, Wielinga B. Assessing Acceptance of Assistive Social Agent Technology by Older Adults: the Almere Model. Int J of Soc Robotics. 2010;2(4):361-375. [doi:10.1007/s12369-010-0068-5]

The *Psychosocial Impact of Assistive Devices Scale (PIADS*)

Reference: Jutai J, Day H. Psychosocial Impact of Assistive Devices Scale (PIADS). Gelderblom GJ, de Witte LP, eds. TAD. 2002;14(3):107-111. [doi:10.3233/TAD-2002-14305]

Qualitative evaluation:

| What impact did the robot have on your health/wellbeing? |
| --- |
| How did you find the robot’s features and services? Did you find them useful? Why do you think so? |
| What do you think are the potential risks and side effects caused by the use of the robot? What can be done to prevent them? |
| Do you think this robot can have an impact on the loneliness/isolation of the users? Why? |
| How much would you be willing to invest to benefit from the robot in your home? Would you prefer to purchase or to rent the robot? In the case of a rent, would it be for a short or long term? |
| In your opinion, what are the ethical issues to be identified and defined before the deployment of these robots in the homes of future users? |
| In your opinion, what skills and knowledge are necessary for a good deployment of these robots in users’ homes? |
| What factors would restrain you from using this type of robot? |
